# Supplementary material for: The genome sequence of the striped dolphin, Stenella coeruleoalba (Meyen, 1833)
Source: Wellcome Open Res. 2024 Dec 18;9:727. [Version 1] doi: 10.12688/wellcomeopenres.23374.1 (PMC11729189; doi:10.12688/wellcomeopenres.23374.1)
Supplement: Peer review history

# Reviewer report: Reviewer response for version 1

**DOI:** 10.21956/wellcomeopenres.25769.r116783

**Published:** 2025-03-12

**Authors:** Peijun Zhang

**Affiliations:** 1 Institute of Deep-sea Science and Engineering, Sanya, China

This article provides a detailed information of the genome sequence from a male striped dolphin. It was well designed and clearly documented.

**I only have some minor concerns on the writing:**

 1) Abstract:  Artiodactyla suggest to replace with Cetacea. Otherwise, Cetartiodactyla is more suitable here than Artiodactyla. Also in the key words.

 2) Abstract: "The genome sequence has a total..." should be  "The assembled genome sequence ...."

 3) Species taxonomy: I am not sure what is the basis for this taxonomy, anyway, please double check following my suggestion 1).

 4) Background:  Rewrite "The striped dolphin (Figure 1) is an abundant pelagic small dolphin, globally distributed in warm-temperate and tropical waters. Global abundance is estimated to be over 2 million, and it is the most abundant dolphin species in the Mediterranean Sea." to "The striped dolphin **, *Stenella coeruleoalba*, **(Figure 1) is an abundant pelagic small dolphin, globally distributed in warm-temperate and tropical waters. Global abundance is estimated to be over 2 million, and it is **one of the** most abundant dolphin species in the Mediterranean Sea."

 5) Background: last section remove "Stenella coeruleoalba"

 6) Table 2: the symbol ** is not marked  in  Table 2, please mark it.

Are sufficient details of methods and materials provided to allow replication by others?

Yes

Is the rationale for creating the dataset(s) clearly described?

Yes

Are the datasets clearly presented in a useable and accessible format?

Yes

Are the protocols appropriate and is the work technically sound?

Yes

Reviewer Expertise:

Marine mammal research, Genomics

I confirm that I have read this submission and believe that I have an appropriate level of expertise to confirm that it is of an acceptable scientific standard.

# Reviewer report: Reviewer response for version 1

**DOI:** 10.21956/wellcomeopenres.25769.r116788

**Published:** 2025-01-23

**Authors:** Annabel Whibley

**Affiliations:** 1 Grapevine Improvement, Bragato Research Institute, Lincoln, New Zealand; 2 The University of Auckland, Auckland, Auckland, New Zealand

Davidson, Morin and colleagues report the genome sequence of the striped dolphin *Stenella ceoruleoalba*. The genome assembly is of good quality, with close to 90% of the contigs scaffolded into chromosome pseudomolecules using HiC evidence. The sample, sequencing and assembly metadata is well documented and uses current best-practice tools for assembly and evaluation.

 The generation of RNAseq data from the necroscopied individual is presented in Table 1 but does not feature in the report (an accompanying gene annotation has not been generated at the time of review). 

 Please note that there is a discrepancy in the BUSCO version reported (version 5.3.2 is stated in Table 4 vs 5.4.3 in the Genome Report text).

 I would also have been interested to learn more about the putative causes of the reduced intensity of the Hi-C contact map signals for chromosome 7.

Are sufficient details of methods and materials provided to allow replication by others?

Yes

Is the rationale for creating the dataset(s) clearly described?

Yes

Are the datasets clearly presented in a useable and accessible format?

Yes

Are the protocols appropriate and is the work technically sound?

Yes

Reviewer Expertise:

Genomics, Bioinformatics, Evolution

I confirm that I have read this submission and believe that I have an appropriate level of expertise to confirm that it is of an acceptable scientific standard.

# Reviewer report: Reviewer response for version 1

**DOI:** 10.21956/wellcomeopenres.25769.r116786

**Published:** 2025-01-13

**Authors:** Takushi Kishida

**Affiliations:** 1 Nihon University, Fujisawa, Japan

This study provides a high-quality chromosomal-level striped dolphin haploid genome assembly. I have no complaints about the quality of this genome data.

 However, I have some comments about the specimen. The authors do not provide a specimen voucher, but if possible, I strongly recommend that the authors deposit the specimen in an appropriate museum so that the genome data can be associated with morphological data. Otherwise, detailed morphological data, such as 3D scan data, would be appreciated.

Are sufficient details of methods and materials provided to allow replication by others?

Partly

Is the rationale for creating the dataset(s) clearly described?

Yes

Are the datasets clearly presented in a useable and accessible format?

Yes

Are the protocols appropriate and is the work technically sound?

Yes

Reviewer Expertise:

genetics

I confirm that I have read this submission and believe that I have an appropriate level of expertise to confirm that it is of an acceptable scientific standard.

# Reviewer report: Reviewer response for version 1

**DOI:** 10.21956/wellcomeopenres.25769.r116006

**Published:** 2024-12-31

**Authors:** Jayan Duminda M. Senevirathna

**Affiliations:** 1 Uva Wellassa University, Badulla, Uva Province, Sri Lanka

The article is nicely written. However, some mistakes are are noticed as follows;

 - In abstract first sentence, a comma should be placed after the common name of the dolphin. 

 - Scientific name should be italic in keywords

 - In Table 2, ** has not indicated in the table

 - In Table 3, second column should be Chromosome name/number

 - In assembly curation, if the the curation process article is still in preparation, maybe no need to mention.

 - use of RNA seq data has never been mentioned or the results.

Are sufficient details of methods and materials provided to allow replication by others?

Yes

Is the rationale for creating the dataset(s) clearly described?

Yes

Are the datasets clearly presented in a useable and accessible format?

Yes

Are the protocols appropriate and is the work technically sound?

Yes

Reviewer Expertise:

Aquatic Biotechnology and Genetics

I confirm that I have read this submission and believe that I have an appropriate level of expertise to confirm that it is of an acceptable scientific standard.
